# Reperfusion Injury: How Can We Reduce It by Pre-, Per-, and Postconditioning

**DOI:** 10.3390/jcm13010159

**Published:** 2023-12-27

**Authors:** Maria Buske, Steffen Desch, Gerd Heusch, Tienush Rassaf, Ingo Eitel, Holger Thiele, Hans-Josef Feistritzer

**Affiliations:** 1 Department of Cardiology, Heart Center Leipzig at University of Leipzig and Leipzig Heart Science, 04289 Leipzig, Germany; maria.buske@helios-gesundheit.de (M.B.); steffen.desch@medizin.uni-leipzig.de (S.D.); 2 Institute for Pathophysiology, West German Heart and Vascular Center, University of Essen Medical School, 45122 Essen, Germany; gerd.heusch@uk-essen.de; 3 Department of Cardiology and Vascular Medicine, West German Heart and Vascular Center, University Hospital Essen, 45147 Essen, Germany; tienush.rassaf@uk-essen.de; 4 Medical Clinic II, Clinic for Cardiology, Angiology and Intensive Care Medicine, University Heart Center Lübeck, 23538 Lübeck, Germany; ingo.eitel@uksh.de; 5 German Center for Cardiovascular Research (DZHK), Partner Site Hamburg/Kiel/Lübeck, 23538 Lübeck, Germany

**Keywords:** ischemia-reperfusion injury, acute myocardial infarction, infarct size, myocardial injury, ischemic conditioning, percutaneous coronary intervention

## Abstract

While early coronary reperfusion via primary percutaneous coronary intervention (pPCI) is established as the most efficacious therapy for minimizing infarct size (IS) in acute ST-elevation myocardial infarction (STEMI), the restoration of blood flow also introduces myocardial ischemia-reperfusion injury (IRI), leading to cardiomyocyte death. Among diverse methods, ischemic conditioning (IC), achieved through repetitive cycles of ischemia and reperfusion, has emerged as the most promising method to mitigate IRI. IC can be performed by applying the protective stimulus directly to the affected myocardium or indirectly to non-affected tissue, which is known as remote ischemic conditioning (RIC). In clinical practice, RIC is often applied by serial inflations and deflations of a blood pressure cuff on a limb. Despite encouraging preclinical studies, as well as clinical studies demonstrating reductions in enzymatic IS and myocardial injury on imaging, the observed impact on clinical outcome has been disappointing so far. Nevertheless, previous studies indicate a potential benefit of IC in high-risk STEMI patients. Additional research is needed to evaluate the impact of IC in such high-risk cohorts. The objective of this review is to summarize the pathophysiological background and preclinical and clinical data of IRI reduction by IC.

## 1. Introduction

Over the last decades, treatment of acute myocardial infarction (AMI) has markedly improved, with a resulting decrease in mortality and morbidity [1]. Nevertheless, according to the World Health Organization (WHO), ischemic heart disease was still the leading cause of death globally in 2019. Appropriate management is, therefore, crucial to further reduce mortality after AMI [2].

In ST-elevation myocardial infarction (STEMI), reducing infarct size (IS) is a leading treatment goal to improve clinical outcomes [3]. The most effective way to achieve this is through rapid coronary reperfusion by a primary percutaneous coronary intervention (pPCI). However, the restoration of blood flow itself can also contribute to myocardial injury and cardiomyocyte death [4], accounting for up to 50% of the total amount of irreversible myocardial damage in animal studies [5]. This phenomenon, known as myocardial ischemia-reperfusion injury (IRI), was first described by Jennings et al. in 1960 [6]. Since then, various potential underlying mediators have been identified, including oxidative stress, increased intracellular Ca^2+^ concentration, rapid restoration of physiologic pH [7], complement activation with the migration of proinflammatory agents, like interleukins, neutrophils, and cell-adhesion molecules [8], and mitochondrial channel-opening [9]. They include cardiomyocytes or non-cardiomyocytes, such as platelets, leukocytes, smooth muscle cells, or fibroblasts [10], and regulatory mechanisms, such as long non-coding RNA and MicroRNA [11]. However, the exact mechanisms have not been fully understood yet.

Numerous approaches have been explored to mitigate IRI, encompassing mechanical and thermal stress interventions, and various pharmacological approaches [12]. The aim of this review is to provide an up-to-date summary of the latest evidence pertaining to mechanical strategies.

## 2. Local Ischemic Conditioning: Pathophysiological Background and Preclinical Studies

Local ischemic conditioning, in contrast to remote conditioning, means that the protective stimulus is applied directly to the organ or tissue that should be protected.

In brief, the principle is to apply repetitive cycles of ischemia and reperfusion. Depending on the time point of conditioning relative to definite reperfusion, ischemic conditioning is further classified as pre-, per-, or postconditioning [5].

The protective mechanisms of local ischemic preconditioning and postconditioning have been reported to share the activation of certain pathways, such as the reperfusion-injury salvage kinase (RISK) pathway, the survival-activating factor enhancement (SAFE) pathway [13,14], and the JAK-STAT pathway [15], as well as cause the inhibition of mitochondrial permeability transition pore (MPTP) formation [16]. It is also known that ischemic postconditioning inhibits inflammatory responses, potentially through reduced synthesis and the secretion of tissue necrosis factor alpha (TNFα) and interleukin-6 (IL-6) [17].

Local ischemic preconditioning was first applied by Murry et al. in 1986, who showed that four cycles of 5 min coronary occlusion followed by 5 min of reperfusion prior to definite coronary occlusion markedly reduced IS in dogs [18]. This protective effect was confirmed by Li et al. only a few years later, who applied multiple occlusions of the left circumflex coronary artery (LCX), each lasting five minutes, followed by a sustained 60 min occlusion of the same vessel, showing a significant reduction in IS compared to the group with only a sustained occlusion [19].

Local ischemic postconditioning first was applied in anesthetized dogs, with three cycles of 30 s occlusion followed by 30 s of reperfusion starting immediately after reperfusion after 60 min of complete coronary occlusion. The authors showed a 44% decrease in IS using postconditioning compared to the control group [20]. Similar findings were also made in other experimental studies in rabbits (68% increase in salvage) and pigs (52% increase in salvage) [21].

## 3. Remote Ischemic Conditioning: Pathophysiological Background and Preclinical Studies

In contrast to local ischemic conditioning, remote ischemic conditioning (RIC) involves additional pathways that transmit the cardioprotective stimulus from distant tissues to the myocardium [13,22,23]. Although the precise mechanism has not been fully understood yet, it is known to involve the activation of peripheral sensory nerves, as well as the neuronal and humoral transfer of a cardioprotective signal from the site of stimulus to the target organ [13,24].

In the field of RIC, two major concepts exist. The first is intracardiac RIC, in which preceding ischemia/reperfusion is applied to a myocardial region distant from the subsequent sustained ischemia zone. Following the same conditioning regimen as Murry et al., Przyklenk et al. were the first to apply remote preconditioning to a coronary artery distant from the culprit lesion, demonstrating a significant reduction in resulting IS [25]. The clinical applicability of intracardiac conditioning is limited because of its invasive character. Consequently, the second concept with higher applicability in practice is interorgan RIC, where ischemia/reperfusion is induced in an organ or tissue far from the heart itself [22]. In both cases, antecedent brief episodes of myocardial ischemia protect remote, virgin myocardium from subsequent sustained ischemia in canine experiments [25]. Interorgan RIC can encompass various organs and tissues, with intermittent renal and limb ischemia applied in several pivotal studies [25,26,27]. Regardless of whether a pre-, per-, or postconditioning strategy was applied, a reduction in IS has been demonstrated with RIC in different animal models [28,29,30,31,32]. Kharbanda et al. first translated the concept to humans by demonstrating that RIC of the upper limb conferred protection against endothelial dysfunction induced by IRI [33].

## 4. Effect of Ischemic Conditioning on Myocardial Injury in STEMI Patients

**Figure 1:**
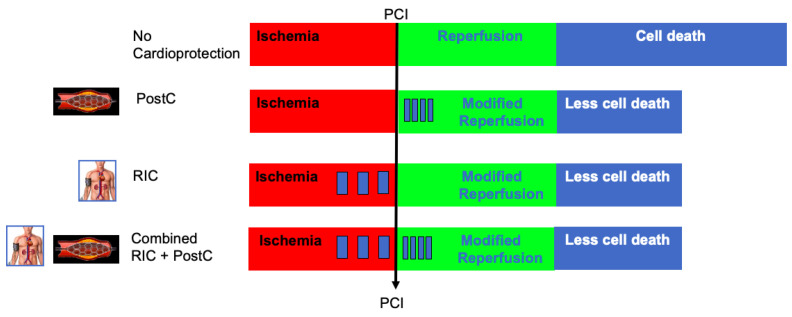
Timing and application of ischemic conditioning for cardioprotection in acute myocardial infarction. PostC: Local Ischemic Postconditioning; RIC: Remote Ischemic Conditioning.

In a clinical setting, local ischemic postconditioning and RIC are often combined, aiming to enhance cardioprotective effects (Figure 1).

Studies investigating the effect of ischemic conditioning on myocardial injury in patients with STEMI are summarized in Table 1.

Unlike the consistent outcomes observed in animal models, research on humans has yielded contradictory findings.

Staat et al. performed a randomized study of local ischemic postconditioning in 30 STEMI patients. Four repetitive cycles of 1 min inflation and 1 min deflation of the angioplasty balloon starting within 1 min of reperfusion led to a 36% decrease in enzymatic IS as determined by total CK release at 72 h compared to the control group [35]. A reduction in enzymatic IS using local ischemic conditioning could be confirmed by numerous subsequent prospective studies with a larger number of patients [36,39,69]. Only one small, randomized study failed to show the benefit of local ischemic postconditioning on enzymatic IS in STEMI patients. However, this study, exclusively including patients undergoing manual thrombectomy, started local ischemic postconditioning 5 min after reflow, which might have impacted the results [41].

Numerous trials demonstrated a decrease in IS, evaluated through enzyme release, with the use of RIC alone or combined with local ischemic postconditioning [34,36,37,38,40,48]. In a clinical setting, RIC can be achieved by serial inflations and deflations of a blood pressure cuff applied to a limb. For example, Crimi et al. showed a 12% reduction in enzymatic IS in STEMI patients when applying three cycles of 5 min inflation and deflation of a standard blood pressure cuff on the upper arm [38]. In the RIPOST-MI trial, Prunier et al. used a combination of RIC and local ischemic postconditioning and showed a 29% reduction in IS as assessed by CK-MB at 72 h compared to the control group [36]. Even in patients undergoing thrombolysis for STEMI, Yellon et al. found a significant reduction in IS, as indirectly assessed by CK-MB and Tn-I release using RIC [34].

Numerous clinical studies of a smaller scale also have reported significant reductions in IS as assessed by single-photon emission computed tomography (SPECT) at various time points, including 72 h, 3 days, and 6 months [42,43,44,70]. The CONDI-1 trial (*n* = 333), for example, found an improved myocardial salvage index (MSI) by 21% at 30 days as assessed by SPECT when using RIC as an adjunct to pPCI for STEMI patients [43]. The effect was strongest in patients with totally occluded vessels and infarction of the left anterior descending artery (LAD) [43].

However, SPECT is a relatively gross measurement of IS compared to cardiac magnetic resonance (CMR), which has proven to be superior to SPECT with regard to the detection and quantification of AMI [71]. When assessed by CMR, we encounter conflicting findings concerning the impact of ischemic conditioning. Numerous studies have demonstrated the potential for ischemic conditioning to yield a reduction in IS or improvement in MSI over follow-up periods spanning from 2 days to as long as 12 months [44,45,47,48,49,53,55]. As an illustration, Araszkiewicz et al. found a 47% decrease in IS, evaluated by CMR, among STEMI patients when employing local ischemic postconditioning compared to the control group after 48–96 h [47]. The LIPSIA CONDITIONING trial also showed a significant decrease in MSI among STEMI patients when applying a combination of RIC and local ischemic postconditioning. However, when utilizing local ischemic postconditioning alone, no significant reduction was observed compared to standard treatment [48].

Some other studies also did not find any beneficial effect of local ischemic postconditioning or RIC regarding CMR endpoints in STEMI patients [56,58,61,63,65,66]. For example, Sörensson et al. did not find significant differences in the MSI between the control and postconditioning group when assessed by CMR performed 6–9 days after STEMI [61].

An important observation of these studies is that both RIC and local ischemic postconditioning were feasible and safe. The study regimen induced no complications in the coronary arteries (e.g., coronary artery dissection, stent deformation, acute re-occlusion) or in the conditioned limb, such as thrombophlebitis. Furthermore, the patients tolerated the procedure well, with no adverse events reported during the procedures or during follow-up.

## 5. Effect of Ischemic Conditioning on Clinical Outcomes in STEMI Patients

Studies on the clinical outcome of both RIC and local ischemic postconditioning have shown disappointing results, as summarized in Table 2.

The POST trial included 700 patients and showed no reduction in the combined endpoint of death, myocardial infarction, severe heart failure, or stent thrombosis at 12 months, using local ischemic postconditioning compared to standard care [78]. The DANAMI-3-iPOST trial (*n* = 1234) also showed no reduction in all-cause death and hospitalization for heart failure at a mean follow-up of 38 months with local ischemic postconditioning [79]. The extended follow-up study of the DANAMI-3-iPOST trial showed a long-term clinical benefit of local ischemic postconditioning. After a mean follow-up of 4.8 years, there was a significant reduction in the combined endpoint of cardiovascular mortality and hospitalization for heart failure, but only in the PCI-only subgroup (without thrombectomy) (15% vs. 22%, *p* = 0.023) [74]. The LIPSIA CONDITIONING trial, testing both local ischemic postconditioning alone and in combination with RIC, failed to reduce the combined endpoint of cardiac death, reinfarction, and new congestive heart failure at 6 months [72]. In contrast, the long-term follow-up during a median of 3.6 years showed a significant decrease in MACE within the group that underwent combined RIC and local ischemic postconditioning. This reduction was primarily attributed to a significant decrease in new cases of congestive heart failure. However, local ischemic postconditioning alone did not lead to a significant reduction in MACE compared to the control group [72]. The study is also limited by its post hoc nature and limited sample size, which was calculated based on the primary endpoint MSI, rather than clinical events.

The CONDI-2/ERIC-PPCI trial is by far the largest randomized trial testing the effect of RIC on clinical outcomes. In this trial, 5401 STEMI patients were randomized to either RIC prior to pPCI or standard treatment. The primary endpoint was a combination of cardiac death and hospitalization for heart failure at 12 months. The study failed to show an improvement in the primary outcome at 1 year. Furthermore, RIC had no effect on major secondary endpoints, including myocardial IS assessed by cTn T release (although a complete set of high-sensitivity cTn T data was only available for <15% of patients) [77]. The recent registry-based randomized trial by Bainey et al. comparing RIC and standard care in patients with STEMI also failed to show any reduction in MACE or IS or a decrease in LV-EF after 1 year [62]. The long-term follow-up analysis from the CONDI-1 trial (mean long-term follow-up of 3.8 years) showed a 48% relative reduction in MACCE, mainly driven by a reduction in all-cause mortality. However, the trial was powered to detect changes in MSI, not long-term outcomes [73].

To date, no randomized study adequately powered for clinical outcome has demonstrated a significant benefit from RIC or local ischemic postconditioning on clinical outcomes in STEMI patients.

## 6. Discussion

Various approaches have been explored over recent decades to mitigate IS in STEMI patients. In addition to RIC and local ischemic postconditioning, as described in the present article, various other approaches have been tested to reduce myocardial damage. These techniques include left ventricular unloading by an intra-aortic balloon pump or transvalvular microaxial flow pump aiming to reduce myocardial workload and enhance coronary perfusion, potentially limiting IS in STEMI patients (ongoing STEMI-DTU trial; NCT03947619) [80]. Therapeutic hypothermia, as well as a plethora of drugs, like cariporide, ciclosporin A, metoprolol, and adenosine, have been tested to reduce myocardial damage in preclinical and clinical studies [16]. Despite promising results in several pilot studies, translation into improved clinical outcomes could not be confirmed for the majority of these approaches.

Available data indicate that conditioning may be beneficial primarily in a selected cohort of STEMI patients, including those with a pre-pPCI TIMI flow grade of less than 2, no collateralization, high area at risk (AAR), and long delay to reperfusion, all resulting in higher IS [81]. Sörensson et al. showed a significant reduction in IS as assessed by CMR only in the subgroup of patients with an AAR in the upper quartile without a difference in the overall population [66]. Several clinical cardioprotection studies in STEMI patients suggest that patients presenting with occlusion of the right coronary artery (RCA) or LCX, where the resulting IS is relatively small, do not benefit as much from cardioprotective therapy as those presenting with large anterior infarction [35,43,44,67,82,83]. On the other hand, other studies in patients with anterior STEMI did not observe any significant improvement in LV-EF after 6 months or IS after 1 week [59,68]. The implementation of STEMI networks in well-developed medical care systems goes ahead with a reduction in pain-to-device times and, therefore, reduces IS in STEMI patients. For instance, in the CONDI-2-ERIC-PPCI trial, the median pain-to-device time was only three hours. Myocardial damage is smaller in such patients and the prognosis is better, so an additional improvement in clinical outcome by ischemic conditioning may be masked [84]. In the CONDI-2-ERIC-PPCI trial, the IS was relatively small, with a median of 17% of left ventricular mass. In contrast, a small retrospective study suggested that patients with STEMI and a healthcare system delay of more than 120 min may benefit more from RIC than those with a shorter delay [85].

Another attenuating factor is the presence of spontaneous reperfusion before pPCI. Due to the early implementation of effective antiplatelet and antithrombotic therapy, a significant proportion of STEMI patients present with spontaneous reperfusion. For example, approximately 20% of participants in the CONDI-2/ERIC-PPCI study showed spontaneous recanalization upon admission. Pre-specified subgroup analyses in the CONDI-2/ERIC-PPCI trial, however, did not show any significant differences in clinical outcomes with RIC according to pre-PCI TIMI flow grade [86]. Roubille et al. showed that IS reduction by local ischemic postconditioning is lost when applied to patients with a TIMI 2–3 flow grade at admission [87].

The substantial advancements in providing early pPCI, as well as improvements in antiplatelet and antithrombotic therapy, have significantly improved the prognosis of STEMI over the past three decades. Consequently, demonstrating an additional clinical benefit by ischemic conditioning has become increasingly challenging. However, ischemic conditioning might be beneficial in high-risk cases, where mortality is higher. This could result in a higher potential for additional prognostic benefits [88]. Cardioprotective effects of RIC are more pronounced in patients with longer ischemia times [85]. In the LIPSIA CONDITIONING trial, the authors described a trend of higher MSI in the conditioning group compared to standard therapy in patients presenting with Killip class ≥2 on admission [48]. Another observational study demonstrated improved clinical outcomes with RIC in patients with STEMI-related cardiogenic shock and cardiac arrest [76]. Consequently, ischemic conditioning might provide clinical benefits in particular in medical care systems with less well-developed STEMI networks, resulting in longer ischemia times and a suboptimal reperfusion strategy [89]. Therefore, clinical trials testing the effect of ischemic conditioning in high-risk STEMI populations are needed [90]. The ongoing RIC-AFRICA trial is going to investigate the effect of RIC in such a high-risk population with a higher prevalence of cardiovascular risk factors, suboptimal use of secondary prevention, and poor access to early reperfusion with pPCI [91].

The ongoing randomized, multicentric RIP-HIGH trial aims to investigate the effect of combined RIC plus local ischemic postconditioning compared to standard treatment in a high-risk STEMI population presenting with Killip-class ≥2 (NCT04844931). The primary endpoint is a combination of all-cause mortality and heart failure hospitalization at 12 months.

Therefore, a more detailed understanding of the potential benefits of ischemic conditioning through these ongoing trials is expected. They are addressing the limitations of previous trials by focusing on high-risk populations, combined approaches, the use of extended follow-up, and highly relevant clinical endpoints to provide further evidence regarding the clinical efficacy of ischemic conditioning in STEMI.

## 7. Conclusions

IRI poses a notable challenge in the management of STEMI patients undergoing reperfusion therapy. Various strategies have been studied, such as local and remote ischemic pre-, per-, and postconditioning. These approaches have shown promising outcomes in terms of reducing myocardial injury and improving myocardial function, but the translation of these findings into improved clinical outcomes for STEMI patients has failed so far. Further research is required, particularly focusing on high-risk patients, as cardioprotective strategies might be even more effective in the setting of a more severe myocardial injury. 

## Figures and Tables

**Table 1:** Clinical trials of ischemic conditioning and myocardial injury in STEMI patients.

First Author, Year	Patients (IC/Control)	Time of IC	Procedure	Endpoint	Outcome (*p*-Value)
Enzymatic IS					
Yellon et al., 2015 [34] (ERIC-LYSIS)	261/258	initiated before and continued during thrombolysis	RIC upper limb 4 cycles (5/5 min)	IS (CK-MB, TnT) at 24 h	32%/19% reduction (0.026/0.020)
Staat et al., 2005 [35]	58/56	within 1 min of reperfusion	PostC 4 cycles (60/60 s)	IS (CK) at 72 h	36% reduction (<0.05)
Prunier et al., 2014 [36] (RIPOST-MI)	18 (RIC)/20 (RIC + Post)/17	RIC: within minutes of admission to the cath. laboratory PostC: within 1 min of reflow	RIC upper limb 3 cycles (5/5 min) PostC 4 cycles (30/30 s)	IS (CK-MB) at 72 h	RIC: 29% reduction (0.016) RIC + PostC: 36% reduction (0.042)
Cao et al., 2018 [37]	36/33	within 1 min of reperfusion	RIC upper limb 4 cycles (5/5 min)	IS (CK-MB) at 72 h	39% reduction (<0.01)
Crimi et al., 2013 [38]	50/50	started with thrombectomy or balloon inflation	RIC lower limb 3 cycles (5/5 min)	IS (CK-MB) at 72 h	20% reduction (0.043)
Zhao et al., 2009 [39]	25/24/26	after stent implantation	PostC 3 cycles (30/30 s or 60/60 s)	IS (cTnI) at 7 d	25% reduction (<0.05) 57% reduction (<0.05)
Wang et al., 2014 [40]	23/23	started within 1 min after reflow	RIC lower limb 3 cycles (5/5 min)	IS (CK-MB) at 72 h	no difference (>0.050)
Luz et al., 2015 [41]	43/44	within 5 min after reflow	PostC 4 cycles (60/60 s)	IS (TnT) at 72 h	no difference (0.68)
IS assessed by SPECT					
Xue et al., 2010 [42]	23/20	immediately after initial reperfusion	PostC 4 cycles (60/60 s)	IS at 72 h	46% reduction (0.002)
Bøtker et al., 2010 [43] (CONDI-1)	166/167	during ambulance transfer	RIC upper limb 4 cycles (5/5 min)	MSI at 30 d	45% increase (0.033)
Thibault et al., 2008 [44]	16/21	within 1 min of reflow after the direct stenting	PostC 4 cycles (60/60 s)	IS at 6 months LV-EF at 12 months (TTE)	39% reduction (0.040) 7% increase (0.040)
CMR parameters					
Garcia et al., 2011 [45]	22/21	immediately upon crossing the lesion with the guide wire	PostC 4 cycles (30/30 s)	LV-EF at index admission	17% increase (0.050)
Thuny et al., 2012 [46]	25/25	within 1 min of reflow after direct stenting	PostC 4 cycles (60/60 s)	myocardial edema/IS at 2–3 d	32/38% reduction (0.03/0.01)
Araszkiewicz et al., 2019 [47]	37/37	immediately after initial reperfusion	PostC 4 cycles (60/60 s)	IS at 2–4 d	47% reduction (0.007)
Eit el al., 2015 [48] (LIPSIA CONDITIONING)	RIC+ postC + PCI (181) vs. PostC + PCI (198) vs. PCI only (187)	pre: started directly after hospital admission post: within 1 min of reperfusion	pre: RIC upper limb 3 cycles (5/5 min) post: PostC 4 cycles (30/30 s)	MSI at 3 d	22.5% increase RIC + PostC (0.02) no difference in postC vs. control (0.39)
White et al., 2015 [49]	43/40	initiation prior to pPCI	RIC upper limb 4 cycles (5/5 min)	IS at 3–6 d	27% reduction (0.009)
Liu et al., 2016 [50]	59/60	during ambulance transfer	RIC upper limb 4 cycles (5/5 min)	early MVO at 3–7 d	33% reduction (0.011)
Mewton et al., 2013 [51]	25/25	within 1 min of reflow after direct stenting	PostC 4 cycles (60/60 s)	early/late MVO size at 4 d	50/56% reduction (0.02/0.01)
Wang et al., 2022 [52]	165/163	started immediately before pPCI and repeated daily for 30 days after PPCI	RIC upper limb 4 cycles (5/5 min)	MSI 5–7 d	9% increase (0.037)
Lønborg et al., 2010 [53]	59/59	immediately after initial reperfusion	PostC 4 cycles (30/30 s)	IS in relation to AAR at 3 months	19% reduction (<0.010)
Koreneva et al., 2021 [54]	43/44	pre: directly after hospital admission; conducted during pPCI post: 90 min after reopening	pre: RIC upper limb 4 cycles (5/5 min) post: RIC upper arm 4 cycles (5/5 min)	IS at 6 months	26% reduction (0.014)
Traverse et al., 2019 [55]	65/57	immediately after initial reperfusion	PostC 4 cycles (30/30 s)	LVEDV-reduction at 12 months	90% reduction (<0.005)
Tarantini et al., 2012 [56]	39/39	within 1 min of reflow after direct stenting	PostC 4 cycles (60/60 s)	IS at 30 d	trending toward IS reduction (0.054)
Kim et al., 2015 [57] (POST substudy)	56/55	immediately after initial reperfusion	PostC 4 cycles (60/60 s)	MSI at 3 d	no difference (0.86)
Garcia del Blanco et al., 2021 [58] (COMBAT-MI)	102/120	at least 20 min before artery aperture	RIC upper arm 4 cycles (5/5 min)	IS at 3–7 d	no difference (0.827)
Verouhis et al., 2016 [59] (RECOND)	47/46	started at arrival at cath. laboratory; continued during angioplasty	RIC 1 cycle before revascularization; 4 cycles in total (5/5 min)	MSI at 4–7 d	no difference (0.260)
Bodi et al., 2014 [60]	49/52	1 min after stent deployment	PostC 4 cycles (60/60 s)	IS, MVO size at 6 d	no difference (0.2/0.3)
Sörensson et al., 2010 [61]	38/38	starting 1 min after initial reperfusion	PostC 4 cycles (60/60 s)	MSI at 7 d	no difference (>0.05)
Bainey et al., 2022 [62]	129/123	initiated as soon as possible and continued during pPCI	RIC upper limb 4 cycles (5/5 min)	IS at 90 d	no difference (0.790)
Limalanathan et al., 2014 [63] (POSTEMI)	136/136	starting 1 min after reperfusion	PostC 4 cycles (60/60 s)	IS at 4 months	no difference (0.180)
Vanezis et al., 2018 [64] (DREAM)	38/35	day 3 after pPCI	RIC upper limb 4 cycles (5/5 min) for 28 consecutive days	improvement in LV-EF at 4 months	no difference (0.924)
Verouhis et al., 2021 [65]	47/46	started at arrival at cath. laboratory; continued during angioplasty	RIC lower limb min 1 cycle before revascularization; min. of 4 cycles after (5/5 min)	MSI at 6 months	no difference (0.230)
Sörensson et al., 2013 [66]	33/35	1 min after initial reperfusion	PostC 4 cycles (60/60 s)	MSI and LV-EF at 12 months	no difference (>0.05)
Changes in LV-EF assessed by a TTE					
Munk et al., 2010 [67]	108/110	during ambulance transfer	RIC upper limb 4 cycles (5/5 min)	LV-EF at 30 d	no difference (0.220)
Elbadawi et al., 2017 [68]	36/35	within 1 min of reopening	RIC lower limb 3 cycles (5/5 min)	LV-EF at 6 months	no difference (0.420)
Wang et al., 2022 [52]	165/163	started immediately before pPCI and repeated daily for 30 d after pPCI	RIC upper limb 4 cycles (5/5 min)	LV-EF at 12 months	no difference (0.117)

AAR: area at risk, CK: creatine kinase, CK-MB: creatine kinase–myoglobin band, CMR: cardiac magnetic resonance, cTnI: cardiac Troponin I, IC: ischemic conditioning, IS: infarct size, LV-EF: left ventricular ejection fraction, MSI: myocardial salvage index, MVO: microvascular obstruction, pPCI: primary percutaneous coronary intervention, PostC: local ischemic postconditioning, RIC: remote ischemic conditioning, SPECT: single-photon emission computed tomography TnT: Troponin T, TTE: transthoracic echocardiogram.

**Table 2:** Clinical trials of ischemic conditioning and clinical outcome in STEMI patients.

First Author, year	Patients (IC/Control)	Time of IC	Procedure	Endpoint	Outcome (*p*-Value)
Stiermaier et al., 2019 [72]	RIC+ postC + PCI (232) vs. PostC + PCI (232) vs. PCI only (232)	pre: started directly after hospital admission post: within 1 min of reperfusion	RIC upper limb 3 cycles (5/5 min) PostC: 4 cycles (30/30 s)	combined endpoint of cardiac death, reinfarction, and new HF at 3.6 years	40% reduction RIC + PostC (0.04) no difference PostC alone (0.41)
Sloth et al., 2014 [73]	166/167	during ambulance transfer	RIC upper limb 4 cycles (5/5 min)	MACCE at 3.8 years (death, MI, readmission for HF, IS, TIA)	47% reduction (0.018)
Madsen et al., 2022 [74] (follow-up of DANAMI-3-iPOST	617/617	immediately after initial reperfusion	PostC 4 cycles (30/30 s)	all-cause death and hospitalization for HF at a median of 4.8 years	32% reduction (0.023)
Gaspar et al., 2018 [75] (RIC-STEMI)	231/217	10 min before the estimated balloon inflation	RIC lower limb 3 cycles (5/5 min)	combination of cardiac mortality and hospitalization for HF after 2.1 years	57% reduction (0.010)
Cheskes et al., 2020 [76]	866/801	earliest possible time after a STEMI was identified (either prehospital or ER)	RIC upper limb 4 cycles (5/5 min)	MACE at 90 d	no difference (0.250)
Eitel et al., 2015 [48] (LIPSIA CONDITIONING)	RIC+ postC + PCI (230) vs. PostC + PCI (231) vs. PCI only (230)	pre: started directly after hospital admission post: within 1 min of reperfusion	pre: RIC upper limb 3 cycles (5/5 min) post: PostC 4 cycles (30/30 s)	MACE at 6 months	no difference (0.400)
Hausenloy et al., 2019 [77] (CONDI-2/ERIC-PPCI)	2546/2569	during ambulance transfer or upon arrival at the hospital	RIC upper limb 4 cycles (5/5 min)	composite of cardiac death or hospitalization for HF within 12 months	no difference (0.320)
Bainey et al., 2022 [62]	129/123	initiated as soon as possible and continued during PCI	RIC upper limb 4 cycles (5/5 min)	composite of death, HF, cardiogenic shock, MI at 12 months	no difference (0.110)
Hahn et al., 2015 [78] (POST)	550/550	within 1 min of reperfusion	PostC 4 cycles (60/60 s)	death, MI, severe HF, or stent thrombosis at 12 months	no difference (0.400)
Engstrøm et al., 2017 [79] (DANAMI-3-iPOST	617/617	immediately after initial reperfusion	PostC 4 cycles (30/30 s)	all-cause death and hospitalization for HF at 38 months	no difference (0.660)

HF: heart failure, MACCE: major adverse cardiac and cerebrovascular event, MACE: major adverse cardiac event, MI: myocardial infarction, PostC: local ischemic postconditioning, RIC: remote ischemic conditioning.

## Data Availability

Data sharing not applicable.
